# Targeted RNA-Based Oxford Nanopore Sequencing for Typing 12 Classical HLA Genes

**DOI:** 10.3389/fgene.2021.635601

**Published:** 2021-03-04

**Authors:** Tiira Johansson, Satu Koskela, Dawit A. Yohannes, Jukka Partanen, Päivi Saavalainen

**Affiliations:** ^1^Translational Immunology Research Program and Department of Medical and Clinical Genetics, University of Helsinki, Helsinki, Finland; ^2^Finnish Red Cross Blood Service, Helsinki, Finland

**Keywords:** human leukocyte antigen, HLA genotyping, nanopore sequencing, RNA sequencing, MinION

## Abstract

Identification of human leukocyte antigen (HLA) alleles from next-generation sequencing (NGS) data is challenging because of the high polymorphism and mosaic nature of HLA genes. Owing to the complex nature of HLA genes and consequent challenges in allele assignment, Oxford Nanopore Technologies’ (ONT) single-molecule sequencing technology has been of great interest due to its fitness for sequencing long reads. In addition to the read length, ONT’s advantages are its portability and possibility for a rapid real-time sequencing, which enables a simultaneous data analysis. Here, we describe a targeted RNA-based method for HLA typing using ONT sequencing and SeqNext-HLA SeqPilot software (JSI Medical Systems GmbH). Twelve classical HLA genes were enriched from cDNA of 50 individuals, barcoded, pooled, and sequenced in 10 MinION R9.4 SpotON flow cell runs producing over 30,000 reads per sample. Using barcoded 2D reads, SeqPilot assigned HLA alleles to two-field typing resolution or higher with the average read depth of 1750x. Sequence analysis resulted in 99–100% accuracy at low-resolution level (one-field) and in 74–100% accuracy at high-resolution level (two-field) with the expected alleles. There are still some limitations with ONT RNA sequencing, such as noisy reads, homopolymer errors, and the lack of robust algorithms, which interfere with confident allele assignment. These issues need to be inspected carefully in the future to improve the allele call rates. Nevertheless, here we show that sequencing of multiplexed cDNA amplicon libraries on ONT MinION can produce accurate high-resolution typing results of 12 classical HLA loci. For HLA research, ONT RNA sequencing is a promising method due to its capability to sequence full-length HLA transcripts. In addition to HLA genotyping, the technique could also be applied for simultaneous expression analysis.

## Introduction

The human leukocyte antigen (HLA) complex on the short arm of chromosome 6 (6p21.3) is the most polymorphic region in the human genome with over 26,000 known alleles reported by the IPD IMGT/HLA database (Release 3.41.2^1^). It is extensively studied, and associated with various infectious and autoimmune diseases (Liu et al., 2016; Karnes et al., 2017; Hirata et al., 2019), and transplantation outcomes (Petersdorf et al., 2015). The HLA genes divided into two classes referred to as class I (*HLA-A, -B*, and -*C*) and class II (*HLA-DR, -DQB*, and -*DP*) play a critical role in immune responses (The MHC sequencing consortium, 1999; Horton et al., 2004).

The major challenge in HLA typing is the high genetic heterogeneity, which makes allele-calling complex. Over the past years, next-generation sequencing (NGS) has taken over and replaced the more conventional HLA typing methodologies. Several different sequencing methods and data analysis tools have emerged to provide high-throughput typing of these polymorphic genes (Erlich et al., 2011; Lank et al., 2012; Shiina et al., 2012; Warren et al., 2012; Mayor et al., 2015; Buchkovich et al., 2017; Orenbuch et al., 2020). Many of the NGS technologies for HLA typing are based on the Illumina sequencing platform, which produces short reads with high sequencing accuracy and outstanding throughput. However, the limited read length has been challenging in HLA typing because of the high sequence similarity and numerous polymorphisms and mosaic structure between alleles. For that reason, sequencing platforms producing long reads, such as Oxford Nanopore Technologies (ONT) and Pacific Bio (PacBio), have raised interest. Longer reads might ease the alignment against HLA alleles and less frequently lead to ambiguous typing results. Both of these long-read sequencing platforms have already proved themselves as an option in HLA research. PacBio’s Single Molecule Real-Time DNA sequencing yielded highly accurate typing results in cell lines (Turner et al., 2018) and improved the survival after hematopoietic stem cell transplantation (Mayor et al., 2019). ONT produced accurate typing results in studies using genomic amplicon sequencing (Liu et al., 2018; Ton et al., 2018; Stockton et al., 2020). ONT was also the first reported NGS HLA typing method tested for deceased donor allocation (De Santis et al., 2020). In addition to genomic sequencing data, ONT was used to genotype HLA class I loci and to determine their gene-level expression from RNA sequencing (RNA-Seq) reads (Montgomery et al., 2020).

Oxford Nanopore Technologies’ MinION is a small portable sequencer connected to a computer through a USB 3.0 cable (Quick et al., 2014). It utilizes nanopore sequencing technology and has the ability to sequence both DNA and RNA with the possibility for a simultaneous real-time data analysis. MinION flow cell contains 2,048 membrane-embedded nanopores, which enable the measuring of fluctuations in the ion flow passing through the nanopores. In the past years, ONT’s sequencing chemistries have developed rapidly leading to faster sequencing speeds and higher data yields.

To evaluate the potential of ONT RNA-Seq for HLA typing, we sequenced 12 HLA genes of 50 individuals in 10 multiplexed MinION sequencing runs. We developed a method, which applied a template switching oligo (TSO) in the reverse transcription (RT) of RNA molecules into cDNA. We then amplified the full-length cDNA and enriched HLA amplicons using primers specific for template switching oligo and HLA genes. We pooled the enriched amplicons, indexed them with sample-specific barcodes, and multiplexed several samples and loci together. Finally, we prepared ONT 2D sequencing libraries, sequenced them on MinION R9.4 SpotON flow cells, and performed the sequence analysis by using SeqPilot software. By comparing the resolved HLA genotypes to alleles obtained by Luminex SSO-PCR, we assessed the accuracy of our method.

## Materials and Methods

### Samples and RNA Extraction

Peripheral blood mononuclear cells (PBMC) of 50 healthy blood donors were isolated using Ficoll-Paque^TM^ Plus (GE Healthcare), Dulbecco’s phosphate buffered saline (DPBS) CTSTM (Gibco Life Technologies), fetal bovine serum (FBS, Sigma), and SepMate^TM^-50 tubes according to the manufacturer’s protocol (Stemcell Technologies). Isolated cell counts were determined using NucleoCounter^®^ NC-100^TM^ (chemometec). The use of anonymized PBMCs from blood donors is in accordance with the rules of the Finnish Supervisory Authority for Welfare and Health (Valvira). All RNA was extracted from 1 to 10 × 10^6^ cells using RNeasy Mini kit and Rnase-Free DNAse Set (both Qiagen), and the quantity was measured using The Qubit^TM^ RNA High Sensitivity Assay Kit in Qubit^®^ 2.0 fluorometer (ThermoScientific). The quality of RNA was assessed with an RNA 6000 Pico Kit (Agilent Genomics) in a 2100 Bioanalyzer (Agilent Genomics). Samples with RNA integrity number (RIN) value over 8.0 were used in the library preparation.

### Reverse Transcription and Amplicon Enrichment

Reverse transcription was adapted from Islam et al. (2012); 25 ng of RNA, 1% Triton^TM^ X-100 (Sigma), 20 μM of STRT-V3-T30-VN oligo, 100 μM of DTT (Invitrogen, Life Technologies, Thermo Fisher), 10 mM dNTP (Bioline), 4 U of Recombinant RNase Inhibitor (Takara Clontech), and 1:1,000 The Ambion^®^ ERCC RNA Spike-In Control Mix (Life Technologies, Thermo Fisher) in a total volume of 3 μl were first incubated 3 min at 72°C. 3.7 μl of RT mix with 5x SuperScript first strand buffer (Invitrogen by Thermo Fisher Scientific), 1 M MgCl_2_ (Sigma), 5 M Betaine solution (Sigma), 134 U of SuperScript^®^ II Reverse Transcriptase (Invitrogen by Thermo Fisher Scientific), 40 μM RNA-TSO 10 bp UMI, and 5.6 U of Recombinant RNase Inhibitor was added and the plate was incubated 90 min at 42°C and 10 min at 72°C. The double-stranded cDNA was further amplified in a 50 μl PCR reaction containing 2x KAPA HiFi HotStart ReadyMix (Kapa Biosystems) and 10 μM ImSTRT-TSO-PCR with the following thermocycling parameters: 1 cycle of 3 min at 95°C, 20 cycles of 20 s at 95°C, 15 s 55°C, 30 s at 72°C, and 1 cycle of final elongation of 1 min at 72°C. A restriction reaction using *Sal*I-HH (New England Biolabs) was used to release the 3′ fragments of the cDNA. DNA was quantified using Qubit^TM^ dsDNA High Sensitivity Assay Kit and the size distribution was checked with High Sensitivity DNA Kit (Agilent Genomics). For HLA amplicon enrichment, 3 μl of template cDNA, 10x Advantage 2 PCR buffer, 50x Advantage^®^ 2 Polymerase Mix (Takara, Clontech), 10 mM dNTP (Bioline), 10 μM TSO forward primer, and one of the seven HLA gene-specific reverse primers (*HLA-A/-B/-C, -DRA, -DRB1/-DRB3/-DRB4/-DRB5, -DQA1, -DQB1, -DPA1*, and *-DPB1*) were incubated 30 s at 98°C following 3 cycles of 10 s at 98°C, 30 s at 55°C, 30 s at 72°C and 27 cycles of 10 s at 98°C, 30 s at 71°C, 30 s at 72°C, and final elongation of 5 min at 72°C in a total volume of 15 μl. Two shared primers were used, one to amplify *HLA-A, -B*, and *-C* and the other for *HLA-DRB1, -DRB3, -DRB4*, and *-DRB5*. Supplementary Figure 1 shows the binding sites of HLA gene-specific primers. All oligos were from Integrated DNA Technologies (Supplementary Table 1). To confirm the amplicon lengths and non-specific amplification, four samples were selected from each locus and run on a 2% agarose gel (Bioline) with 10x BlueJuice^TM^ loading dye (Invitrogen by Thermo Fisher Scientific) in 0.5X TBE (Thermo Fisher Scientific) with the GelGreen^TM^ (Biotium). Gels were visualized using the Quick-Load 1kb DNA Ladder (New England Biolabs). PCR amplicons were quantified using the Qubit^TM^ dsDNA High Sensitivity Assay Kit and the fragment sizes were checked with Agilent’s High Sensitivity DNA Kit. For sequencing library preparation, we divided HLA amplicons into two gene pools per sample using a 5 μl of PCR product from each locus. Gene pool 1 contained genes *HLA-A, -B, -C, -DRB1, -DRB3, -DRB4, -DRB5*, and *-DPB1*, and gene pool 2 *HLA-DRA, -DPA1, -DQA1*, and *-DQB1*. Gene pools were cleaned with 0.7X pool volume Agencourt AMPure XP beads (Beckman Coulter), eluted in 15 μl of nuclease-free water, and quantified with the Qubit^TM^ dsDNA High Sensitivity Assay Kit. Finally, the average fragment size distribution of gene pools 1 and 2 was analyzed using Agilent’s High Sensitivity DNA Kit from 10 samples of both pools. Using this information, the molarity of each pool was calculated using the DNA concentration (ng/μl) and the average fragment length (bp) of the gene pool.

### ONT Library Preparation and Sequencing

ONT’s sequencing compatible barcoded fragments were prepared in a PCR reaction 0.5 nM of DNA from gene pools, 2 μl of PCR barcode from the 96 PCR Barcoding Kit (ONT), 50 μl of LongAmp Taq 2x Mix (New England Biolabs), and Nuclease-Free water in a final volume of 100 μl where ONT’s universal tails were used as a template for barcode introducing primers. The PCR was performed in the following conditions: initial denaturation of 3 min at 95°C, following 15 cycles of 15 s at 95°C, 15 s at 62°C, 30 s at 65°C, and a final extension step 3 min at 65°C. A second DNA purification and size selection was done in a 1X beads:DNA ratio by using the Agencourt AMPure XP beads according to the manufacturer’s instructions and eluted in 20 μl of nuclease-free water. After the purification, DNA was quantified with the Qubit^TM^ dsDNA High Sensitivity Assay Kit and barcoded PCR amplicons were pooled with equal molarities in 10 library pools in a total volume of 50 μl each consisting of 10 individuals and either eight loci (gene pool 1) or four loci (gene pool 2). 1 μg of pooled barcoded PCR products was treated with the NEBNext Ultra II End-repair/dA-tailing Module (New England Biolabs) according a Ligation Sequencing Kit 2D (SQK-LSK208) protocol (ONT) using a DNA CS 3.6kb (ONT) as a positive control. A third DNA purification was performed using 1X beads:DNA ratio by using the Agencourt AMPure XP beads following the Ligation Sequencing Kit 2D protocol. ONT sequencing adapters were ligated using NEB Blunt/TA Ligase Master Mix (New England Biolabs) and Adapter Mix and HP Adaptor provided by ONT following a purification step using MyOne C1 Streptavidin beads (Invitrogen by Thermo Fisher Scientific) according to the Ligation Sequencing Kit 2D protocol to capture HP adaptor containing molecules. The libraries were eluted in 25 μl of elution buffer and mixed with running buffer and library loading beads (ONT) prior to sequencing. All 10 libraries were sequenced for 48 h on R9.4 SpotON flow cells (FLO-MIN106) on MinION Mk 1b device using the MinKNOW software (versions 1.1.21, 1.3.24, 1.3.25, and 1.1.30).

### HLA Typing

ONT’s reads were processed using the 2D Basecalling plus barcoding for FLO-MIN106 250 bps workflow (version v1.125) on the cloud-based Metrichor platform (v2.45.5, v2.44.1, ONT) generating 1D template, 1D complement, and 2D reads. The fastq files were extracted from the native FAST5 files using NanoOK (Leggett et al., 2015). ONT 2D reads were used in genotyping of *HLA-A, -B, -C, -DRA, -DRB1, -DQA1, -DQB1, -DPA1*, and -*DPB1* alleles at high-resolution level with SeqPilot software (v.4.3.1, JSI Medical Systems). The software version used IPD-IMGT/HLA Database. 3.27.0. ONT 2D reads were mapped against the regions of interest (ROIs), which are the target HLA genes selected prior to the sequence analysis. For both gene pools, only those genes were selected, which were present in the gene pool. After the sequence analysis, SeqPilot software reported the genotyping result for each gene of the gene pool. Additionally, SeqPilot reported the number of assigned reads, which were the reads mapping to the selected ROIs, and the number of aligned reads, which were the reads passing the quality filter and added to the coverage. For validation of HLA typing, genomic DNA was extracted from PBMC samples by QiaSymphony SP automat (Qiagen) by following the manufacturer’s protocol. The concentration and purity of DNA were measured by NanoDrop ND-1000 (Thermo Fisher Scientific). The reverse SSOP-Luminex technology (Labtype, One Lambda) was used for HLA typing of *HLA-A, -B, -C, -DRB1, -DRB3, -DRB4, -DRB5, -DQA1, -DQB1, -DPA1*, and *-DPB1*. The results were analyzed with the HLA-Fusion software (v.3.2.0-HF1, One Lambda). The concordance rate of the genotyping results between ONT RNA-Seq and Luminex SSO-PCR data was calculated at one-field (allele group) and two-field (allele) resolution level. Luminex SSO-PCR reports the HLA type at one-field resolution with a list of possible alleles as NMDP codes by the National Marrow Donor Program^2^. For two-field resolution level comparison, we assigned the putative HLA alleles from the allele codes based on the alleles’ prevalence in the Finnish population using a Finnish cohort study (Haimila et al., 2013). The HLA alleles present in our dataset of 50 individuals are shown in Supplementary Table 2.

## Results

### ONT Sequencing Statistics

**FIGURE 1:**
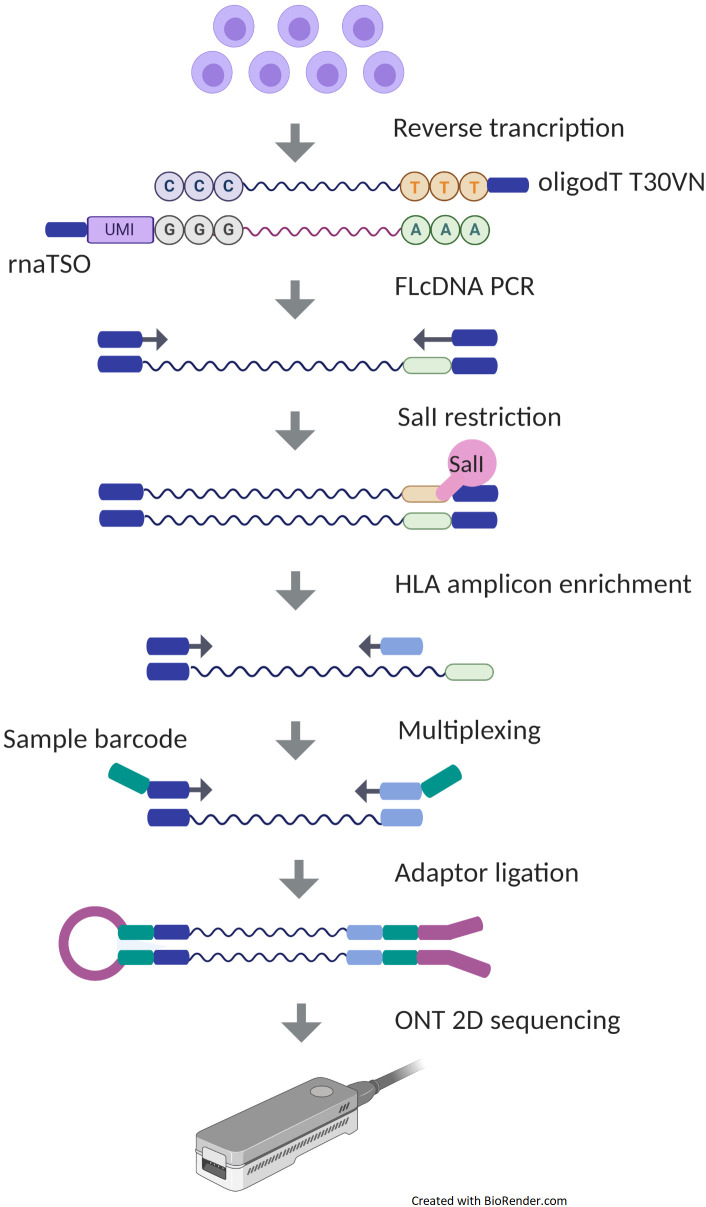
Experimental design of Oxford Nanopore Technologies (ONT) platform. In the sequencing library preparation of ONT, messenger RNA (mRNA) is first reverse transcribed into complementary DNA (cDNA) using an oligodT T30VN primer capturing a polyA tail of mRNA, with simultaneous integration of 10 bp unique molecular identifier (UMI) in RNA template-switching oligonucleotide (rnaTSO), and further amplified using polymerase chain reaction (PCR). The full-length cDNA (FLcDNA) is then processed with *Sal*I restriction enzyme followed by an enrichment of HLA genes and adding sample-specific barcodes for multiplexing. Libraries are ligated with ONT adaptors and sequenced on a MinION sequencer.

**FIGURE 2:**
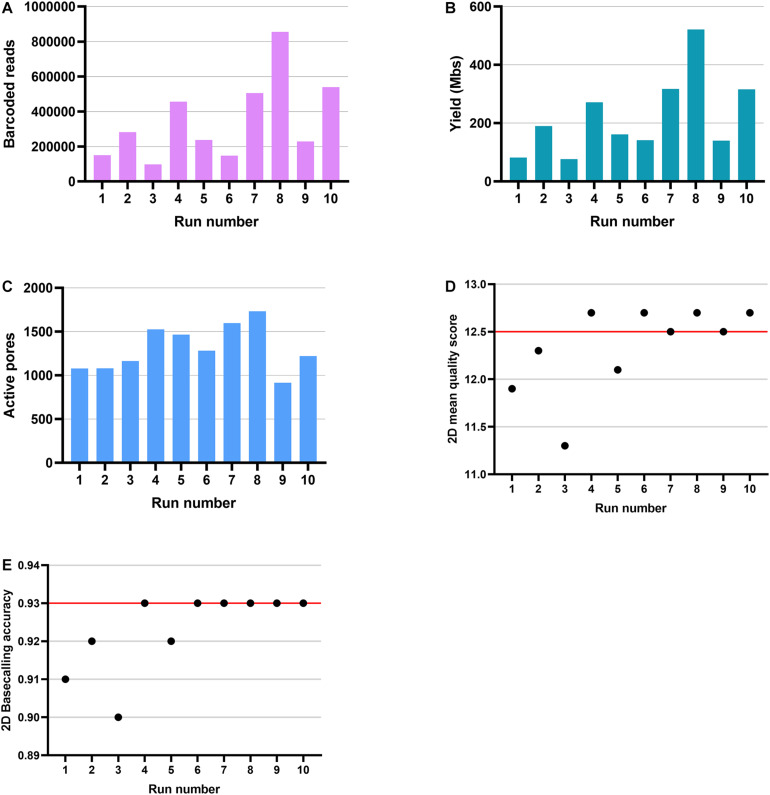
Produced data and quality metrics of ONT MinION sequencing runs. Ten MinION flow cell produced between 97,595 and 855,489 barcoded reads **(A)** and yielded between 76.33 and 521.45 Mbases **(B)**. The number of active pores identified in each run ranged from 915 to 1,732 **(C)**. The distribution for 2D mean quality score was 11.3–12.7 **(D)** and 0.90–0.93 for 2D basecalling accuracy **(E)**. The red lines indicate the median.

We assessed the suitability of ONT RNA-Seq for HLA typing. To do that, we used a targeted 5*′* RNA-Seq method for ONT platform (Figure 1). Simultaneously with the ONT sequencing, cloud-based Metrichor basecalled the raw data and demultiplexed reads per sample-specific barcodes. Metrichor provided both the sequencing statistics and the basecalling results. In the 48-h sequencing runs, 46 h was the maximum time that flow cells were still producing data. However, the majority of the sequence data were generated during the first 8 h. Sequencing of 100 gene pools on 10 ONT MinION SpotON flow cells generated between 97,595 and 855,489 successfully demultiplexed barcoded reads and yielded between 76.33 and 521.45 Mbases per run (Figures 2A,B). An average of 3.4% of reads remained unmultiplexed. All R9.4 flow cells exceeded the guaranteed number of 800 active pores (Figure 2C). Runs produced similar distributions of 2D mean quality scores with a mean of 12.3 (Figure 2D). To assess the quality of basecalling, we used a calibration strand provided in ONT’s Ligation Sequencing Kit 2D. Based on the reads derived from this Lambda genome, the average of 2D basecalling accuracy in 10 runs was 0.92 (Figure 2E). Due to the variation in read lengths, the average read lengths between 520 and 620 bp reported by Metrichor were shorter than the expected amplicon lengths. However, in the sequence length distribution, there were peaks close to the expected amplicon sizes at 800–900 bp for gene pool 1 and at 750–850 bp for gene pool 2.

### Accuracy of ONT RNA-Seq

**FIGURE 3:**
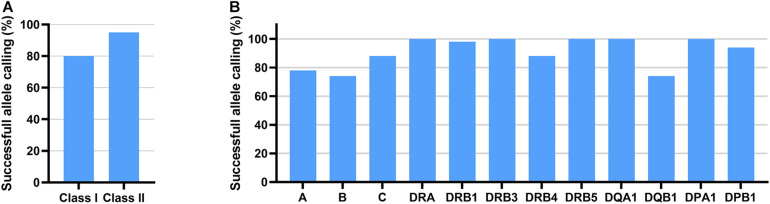
HLA typing results of 50 individuals using ONT RNAseq data and SeqPilot software. Successful allele calling rate is shown for class I and class II **(A)** and for each gene **(B)**.

Oxford Nanopore Technologies’ 2D reads underwent a fastq conversion from FAST5 files with NanoOK tool (Leggett et al., 2015). Extracted fastq files were uploaded to SeqPilot software and aligned against HLA genes selected prior to data analysis. Out of 3,610,894 reads with uniquely identified barcodes, 1,354,704 reads were mapped to ROIs in the SeqPilot software. However, most of the reads uploaded to the software, 62.5%, did not map to the ROIs and remained unmapped. Based on the information received from JSI Medical Systems, this was the first time SeqPilot was used to analyze ONT RNA-Seq data. At the time of the sequence analysis, the software was designed for the analysis of genomic data. This could have possibly contributed to the low mappability of RNA sequencing reads. Using ONT RNA-Seq data, SeqPilot was able to assign 80% of HLA class I alleles and 95% of HLA class II alleles at two-field resolution or higher (Figure 3A and Table 1). HLA typing calls were obtained in 78, 74, and 88% of the cases for *HLA-A, HLA-B*, and *HLA-C* and in 100, 98, 100, 88, 100, 100, 74, 100, and 94% of the cases, for *HLA-DRA, -DRB1, -DRB3, -DRB4, -DRB5, -DQA1, -DQB1, -DPA1*, and *-DPB1* (Figure 3B and Table 1).

**TABLE 1:** HLA genotyping results from ONT RNA-Seq and allele calling rate.

	Result	No result	Accurate allele calling (%)
HLA-A	39	11	78
HLA-B	37	13	74
HLA-C	44	6	88
HLA-DRA	50	0	100
HLA-DRB1	49	1	98
HLA-DRB3*	25	0	100
HLA-DRB4*	15	2	88
HLA-DRB5*	19	0	100
HLA-DQA1	50	0	100
HLA-DQB1	37	13	74
HLA-DPA1	50	0	100
HLA-DPB1	47	3	94

*HLA-DRB3-5 genes are present only in certain haplotypes.

Sequence data analysis in SeqPilot interphase showed an uneven distribution of sequencing coverage for class I genes (Supplementary Figure 2). The coverage graph displayed a lower coverage in exon 1 and 2, and sometimes also in exon 3 area compared to the other exons. For class II, the read coverage was even in all exons. Most of the polymorphisms between HLA alleles lie in exons 2 and 3. Therefore, the low coverage could affect the allele assignment and explain the lower call rates of SeqPilot for class I.

In addition to the coverage graph, we inspected the number of assigned reads mapping to the selected genes. We also calculated the number of aligned reads, which are the reads passing the filter and used in the allele assignment. Table 2 shows the average and range of assigned and aligned reads calculated for each locus. The highest relative ratio between aligned and assigned reads was found in *HLA-DRA, -DRB1, -DQA1*, and *-DPA1* (65–87%), and the lowest in *HLA-DQB1* (22%). In 20% of the cases for class I and in 5% for class II, SeqPilot could not make a confident call. In 76% of these cases for class I and 63% for class II, the number of aligned reads reported by SeqPilot was below the locus average. This suggests that a low sequencing coverage may interfere with successful allele assignment. However, this seems to be true only in some cases since only 32 *HLA-DRB4* reads were sufficient to produce an accurate typing result at three-field resolution.

**TABLE 2:** Summary of assigned and aligned reads per HLA gene from the SeqPilot software.

	Average of assigned reads	Range of assigned reads	Average of aligned reads	Range of aligned reads	Aligned reads (%)
HLA-A	1,271	351–3,551	581	132–1,607	46
HLA-B	1,809	466–6,486	1,013	221–4,156	56
HLA-C	1,501	338–4,516	862	81–2,756	57
HLA-DRA	4,604	1,042–13,897	3,995	873–12,235	87
HLA-DRB1	3,677	1,098–15,607	2,394	672–12,050	65
HLA-DRB3	496	141–1,693	267	44–1,037	54
HLA-DRB4	741	79–2,961	402	32–1,872	54
HLA-DRB5	1,265	132–2,934	747	25–1,815	59
HLA-DQA1	905	179–2,690	692	85–2,263	77
HLA-DQB1	6,734	736–16,924	1,480	119–4,842	22
HLA-DPA1	4,416	803–16,547	3,769	652–14,479	85
HLA-DPB1	1,939	500–7,067	946	222–4,095	49

### Concordance With Luminex SSO-PCR

Human leukocyte antigen typing results obtained from ONT RNA-Seq data were compared to Luminex SSO-PCR genotyping results and the concordance was calculated both at one-field and two-field resolution. Luminex SSO-PCR reports HLA typing result at the allotype level (one-field) with a list of suggested alleles that alter the amino acid sequence (two-field). The first allele in the subtype list is the most common and well-documented one. However, the rare allele subtypes cannot be excluded from the analysis.

**FIGURE 4:**
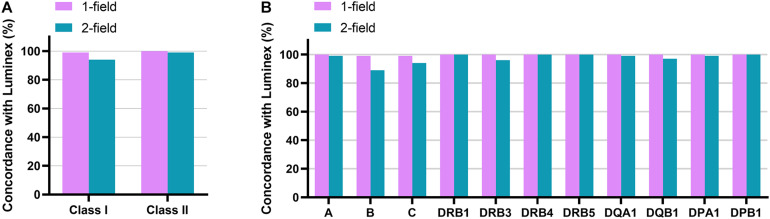
Accuracy of ONT RNAseq method in typing of 12 classical HLA genes. Concordance rate to Luminex SSO-PCR at one-field and two-field typing resolution is shown for class I and class II **(A)** and for each of the 12 genes **(B)**.

ONT RNA-Seq method had an average concordance of 99% (one-field) and 94% (two-field) for class I genes and 100% (one-field) and 99% (two-field) for class II genes with Luminex SSO-PCR results (Figure 4A and Table 3). At one-field level, the results were 100, 99, and 99% concordant for *HLA-A, HLA-B*, and *HLA-C* and 100% concordant for *HLA-DRB1, -DRB3, -DRB4, -DRB5, -DQA1, -DQB1, -DPA1*, and *-DPB1*. A missing second allele in ONT RNA-Seq data caused two one-field resolution discrepancies in one sample for HLA-B and HLA-C between the two methods. Luminex SSO-PCR reported the typing results as *B^∗^07:02, B^∗^35:01* and *C^∗^04:01, C^∗^07:02*. ONT RNA-Seq, however, could only call alleles *B^∗^07:02* and *C^∗^04:01* and reported homozygous results. The number of aligned reads was below the average for both genes. At two-field level, the concordance between ONT RNA-Seq and Luminex SSO-PCR was 99, 89, and 94% for *HLA-A, HLA-B*, and *HLA-C* and 100, 96, 100, 100, 99, 97, 99, and 100% for *HLA-DRB1, -DRB3, -DRB4, -DRB5, -DQA1, -DQB1, -DPA1*, and *-DPB1* (Figure 4B and Table 3). The discrepancy at two-field resolution originated from an allele reported by SeqPilot, which was not present in the list of suggested alleles by Luminex SSO-PCR. The numbers of detected discrepancies were one for *HLA-A, HLA-DQA1*, and *HLA-DPA1*, two for *HLA-DQB1*, four for *HLA-C*, and seven for *HLA-B*. Out of these 16 discrepant samples, 11 (69%) had a lower number of aligned reads compared to the gene average. There were no discrepancies in *HLA-DRB1, -DRB4, -DRB5*, and *DPB1*.

**TABLE 3:** Concordance of HLA typing results between ONT RNA-Seq and Luminex SSO-PCR.

	Concordant results		Disconcordant results		Concordance rate %	
	One-field	Two-field	One-field	Two-field	One-field	Two-field
HLA-A	78	77	0	1	100	99
HLA-B	73	66	1	8	99	89
HLA-C	87	83	1	5	99	94
HLA-DRB1	98	98	0	0	100	100
HLA-DRB3*	25	24	0	1	100	96
HLA-DRB4*	15	15	0	0	100	100
HLA-DRB5*	19	19	0	0	100	100
HLA-DQA1	100	99	0	1	100	99
HLA-DQB1	74	72	0	2	100	97
HLA-DPA1	100	99	0	1	100	99
HLA-DPB1	94	94	0	0	100	100

*For HLA-DRB3-5, results are presented at the sample level.

For class I, there were eight ambiguities in the SeqPilot results at three-field and one at two-field typing resolution. The only ambiguous result at two-field level was in HLA-B. In this case, SeqPilot could not make a confident call between *B^∗^15:01:01G, ^∗^35:01:01G* and *B^∗^15:20, ^∗^35:43:01*. For class II, the sequence analysis resulted in 19 ambiguities at two-field resolution; one for *HLA-DRB5* and *HLA-DQB1*, seven for *HLA-DQA1*, and 10 for *HLA-DPB1*. In nine cases (47%) with ambiguous result, all alleles listed by SeqPilot software were also found in the list of alleles of Luminex SSO-PCR. However, in 10 cases (53%) with ambiguity, SeqPilot reported at least one allele, which Luminex SSO-PCR did not have in its list. In all 15 samples carrying *HLA-DRB4* gene, the possibility of a null allele could not be excluded. There were no ambiguities in *HLA-DRA, -DRB1, -DRB3*, and *-DPA1*.

## Discussion

There are several potential benefits, which ONT offers to HLA research. The long-read single-molecule sequencing technology enables the sequencing of full-length HLA transcripts increasing the possibility of unambiguous phasing in the data analysis step and provides information of the full haplotype block structure. In contrast to short sequencing reads, with ONT’s long reads, smaller sequencing depth may suffice to produce accurate HLA typing results. This enables the multiplexing of several samples making the method cost-effective to use. Additionally, ONT’s portable desktop sequencer, MinION, can be easily adopted in basic laboratory without the need to use sequencing core facilities. Furthermore, the possibility for real-time data analysis during the sequencing may be beneficial for cases where faster HLA typing results are required. An additional benefit could be simultaneous HLA typing and expression determination. Differential expression levels of HLA have been shown to affect numerous human diseases (Apps et al., 2013; Sillé et al., 2013; Schuster et al., 2017; Ramsuran et al., 2018) and to have an impact to the outcome of hematopoietic stem cell transplantation (HSCT) (Petersdorf et al., 2014, 2020). However, despite of these findings, expression analysis is not included in donor selection. With the UMIs already incorporated in our library preparation process, ONT RNA-Seq method could in the future be expanded to enable parallel HLA genotyping and accurate expression quantification using molecule counting.

The main goal of this study was to evaluate the suitability of ONT RNA-Seq for HLA typing. The method included transcription of 25 ng of RNA into cDNA using a template switching oligo, incorporation of a 5′end 10 bp UMI, followed by HLA amplicon enrichment using 5′ end primer specific for template switching sequence and seven 3′ end HLA gene specific primers. To make the approach cost effective, HLA amplicons were divided into two gene pools, and indexed with unique sample-specific barcode sequences. Final steps of the workflow were multiplexing of gene pools of 10 individuals, library preparation using the 2D sequencing chemistry, and sequencing of gene pools on the MinION R9.4 SpotON flow cell. The method enabled sequencing of 10 individuals and eight or four HLA genes, depending on the gene pool, together in a single sequencing run.

The sequence analysis was conducted with SeqPilot software, which has previously been utilized also in the genotyping of HLA-B alleles in genomic amplicon data (Ton et al., 2018). The analysis consisted of two main steps. First, SeqPilot aligned 2D reads against selected HLA genes, which are called ROIs. SeqPilot calculated the number of reads aligning to these selected genes. Second, it performed a quality filtering to the aligned reads. The ones, which passed this step, were used in the allele assignment. After the sequence analysis, SeqPilot reported both the number of aligned reads and the number of assigned reads passing the quality filter. Using ONT RNA-Seq data, SeqPilot software aligned in average of 30,000 reads per individual to the ROIs of both gene pools. Of these reads, approximately 17,000 were good-quality reads, which passed the filter and took part in the allele assignment. At the time of this study, HLA typing software SeqPilot had been validated only for genomic data. Therefore, our study was the first one to use SeqPilot in the analysis of ONT RNA-Seq data. From ONT RNA-Seq reads, SeqPilot successfully called 80% of class I alleles and 95% of class II alleles at two-field resolution or higher. The gene-level allele calling rates were 78, 74, and 88% for *HLA-A, HLA-B*, and *HLA-C.* The low accuracy in these genes might have been caused by the low sequencing depth in exons 1, 2, and 3 we saw in the SeqPilot interphase (Supplementary Figure 1). We are uncertain of what could have caused the lower sequencing depth in this specific area. These are, however, the first three exons of class I genes. For this reason, one possible explanation could be an inadequate efficacy of the RT enzyme to transcribe longer class I molecules. Since most of the allele distinguishing polymorphisms in HLA genes are located in exons 2 and 3, the coverage of these polymorphic positions might not have been sufficient in our ONT RNA-Seq data. This most likely has led to the poorer sensitivity in allele assignment in class I compared to class II genes.

In the majority of cases where SeqPilot could not make a confident call in HLA typing, the number of reads was lower than the average read count with successful calls. Other studies using ONT genomic data have also shown that enough coverage is required for accurate genotyping results (Liu et al., 2018; De Santis et al., 2020; Stockton et al., 2020). However, in some cases, very few reads were sufficient to produce accurate genotyping result. This indicated that there were also other factors affecting the accurate HLA typing such as the sequence read quality and basecalling accuracy. In the SeqPilot interphase, most of the mismatches to the reference allele were caused by a background noise, deletions, or homopolymer regions. With R9.0 MinION flow cells, the rates of miscalls, insertions, and deletions were 6.2, 3.1, and 5.7%, respectively (Jain et al., 2017). Since our study, ONT has launched two new flow cells (R10 and R10.3) and replaced the 2D sequencing chemistry with the 1D^2^ chemistry^3^, which no longer uses the hairpin to physically connect the template and complement strands. However, similarly to ONT’s 2D chemistry, in the 1D^2^ chemistry, both strands of the DNA duplex are sequenced consecutively to obtain an accurate consensus sequence. According to ONT, the quality of 1D^2^ and the number of reads produced should be higher compared to the 2D chemistry. These improvements might provide a solution to the error rate and homopolymers leading to higher allele calling rates and fewer ambiguities in ONT RNA-Seq data.

In the comparison between ONT RNA-Seq and Luminex SSO-PCR, the average concordance at two-field typing resolution was 94% for class I genes and 99% for class II genes. Our results showed that *HLA-B* in ONT RNA-Seq was particularly difficult for SeqPilot to interpret. In all cases with discrepancy in *HLA-B* between the two methods, SeqPilot suggested a more uncommon allele option. The discrepancies were focused to allotypes *B^∗^07* and *B^∗^35*. Compared to the previous studies using MinION in the sequencing of *HLA-B* (Ton et al., 2018; De Santis et al., 2020), our ONT RNA-Seq method did reach as high concordance with the validation method. This might be due to methodological differences or differences in data analysis since both of the previous studies were based on the analysis of genomic data. Additionally, the low sequencing coverage in exons 1, 2, and 3 most likely decreased the class I typing accuracy and hence further affected the concordance rate.

While SeqPilot’s performance in allele calling was promising, the software was not optimized for ONT RNA-Seq data at the time of this study. Several HLA typing algorithms for Illumina RNA-Seq already exist, such as seq2HLA (Boegel et al., 2012), HLAProfiler (Buchkovich et al., 2017), and arcasHLA (Orenbuch et al., 2020). However, the HLA typing tool set for ONT RNA-Seq has remained very limited, and additional HLA typing software dedicated for MinION data would be beneficial. For genomic ONT amplicon data, some HLA genotyping algorithms have already been developed (Liu et al., 2018; Schöfl et al., 2018). However, they have been limited to only certain HLA genes. In addition to these academic algorithms, another commercial HLA typing software, NGSengine (GenDX), for ONT genomic data has been validated recently (van Deutekom et al., 2017). Despite the very promising results obtained with these software, the high error rate of 10–15% (Jain et al., 2017, 2018) occurring in ONT reads and the homopolymer issue (Liu, 2020) are still hampering accurate allele assignment and preventing ONT’s routine applicability for clinical HLA typing. ONT is still a relatively novel technology in HLA research, and the constantly changing sequencing chemistries and high error rates might have hindered the development of comprehensive HLA typing tools. Our study was the second reported work to perform HLA typing from ONT RNA-Seq data and the first where both class I and II genes were included. With the growing interest toward ONT’s long read technology and ONT’s ability to perform accurate HLA typing, we expect the number of bioinformatics tools to grow in the future.

Oxford Nanopore Technologies has already provided direct sequencing from RNA without the need for any amplification steps included in the sequencing library preparation^4^. This is definitely an interesting option and may enable simultaneous HLA typing and HLA expression quantification. With increasing data yields, it might be possible to determine accurately HLA expression at both gene and allele levels. With no additional amplification steps in the library preparation protocol, the workflow would accelerate and eliminate possible PCR bias. While there are many advantages for ONT to be utilized in HLA research, several challenges such as the high error rate and the lack of optimized HLA typing software for ONT data need to be addressed. In addition to the updates in the sequencing chemistry, ONT’s improved nanopore technology of R10.3 flow cells may further lower error rates and result in higher data yields in the future. With improved read accuracy and the homopolymer issue solved, we can also expect more accurate HLA allele calling in ONT RNA-Seq.

In summary, we developed a highly multiplexed RNA-based assay for ONT that enabled genotyping of 12 classical HLA genes of 50 individuals in 10 MinION runs. With the available and future developments in the flow cells and associated sequencing chemistries, we expect the accuracy of the ONT RNA-Seq to improve further. Our method is applicable to the typing of 12 HLA loci in various tissues. With the higher read counts, it may also be used to multiplex even more samples or genes together in a single MinION sequencing run.

## Data Availability Statement

The datasets presented in this study can be found in online repositories. The names of the repository/repositories and accession number(s) can be found below: The European Genome-phenome Archive (EGA), EGAS00001004918.

## Ethics Statement

The studies involving human participants were reviewed and approved by the Finnish National Supervisory Authority for Welfare and Health. Written informed consent for participation was not required for this study in accordance with the national legislation and the institutional requirements.

## Author Contributions

TJ, PS, and JP designed the study and interpreted the results. TJ managed all the samples, prepared the sequencing libraries, performed the sequencing runs, and wrote the manuscript. SK managed Luminex HLA typing. DY extracted the ONT fastq data. TJ analyzed and interpreted the data. All authors read and approved the final manuscript.

## Conflict of Interest

The authors declare that the research was conducted in the absence of any commercial or financial relationships that could be construed as a potential conflict of interest.

## Footnotes

1 https://www.ebi.ac.uk/ipd/imgt/hla/

2 https://bioinformatics.bethematchclinical.org

3 https://nanoporetech.com/about-us/news/1d-squared-kit-available-store-boost-accuracy-simple-prep

4 https://nanoporetech.com/applications/rna-sequencing

## Supplementary Material

Supplementary Figure 1 Binding sites of HLA gene-specific primers.

Supplementary Figure 2 Sequencing coverage graph for class I and class II in the SeqPilot interphase.

Supplementary Table 1 List of primer sequences.

Supplementary Table 2 Alleles of 12 classical HLA genes of 50 individuals.
