# Supplementary material for: Targeted RNA-Based Oxford Nanopore Sequencing for Typing 12 Classical HLA Genes
Source: Front Genet. 2021 Mar 4;12:635601. doi: 10.3389/fgene.2021.635601 (PMC7982845; doi:10.3389/fgene.2021.635601)
Supplement: Supplementary Figure 1 — Binding sites of HLA gene-specific primers. [file Data_Sheet_1.docx]

**Supplementary Figure S1**

**HLA:HLA00001 A*01:01:01:01 1098 bp**

**HLA-ABC gene-specific primer (amplicon length 1074 bp)**

HLA-A*01:01:01:01 ATGGCCGTCATGGCGCCCCGAACCCTCCTCCTGCTACTCTCGGGGGCCCTGGCCCTGACC 60

HLA-ABC ------------------------------------------------------------ 0

HLA-A*01:01:01:01 CAGACCTGGGCGGGCTCCCACTCCATGAGGTATTTCTTCACATCCGTGTCCCGGCCCGGC 120

HLA-ABC ------------------------------------------------------------ 0

HLA-A*01:01:01:01 CGCGGGGAGCCCCGCTTCATCGCCGTGGGCTACGTGGACGACACGCAGTTCGTGCGGTTC 180

HLA-ABC ------------------------------------------------------------ 0

HLA-A*01:01:01:01 GACAGCGACGCCGCGAGCCAGAAGATGGAGCCGCGGGCGCCGTGGATAGAGCAGGAGGGG 240

HLA-ABC ------------------------------------------------------------ 0

HLA-A*01:01:01:01 CCGGAGTATTGGGACCAGGAGACACGGAATATGAAGGCCCACTCACAGACTGACCGAGCG 300

HLA-ABC ------------------------------------------------------------ 0

HLA-A*01:01:01:01 AACCTGGGGACCCTGCGCGGCTACTACAACCAGAGCGAGGACGGTTCTCACACCATCCAG 360

HLA-ABC ------------------------------------------------------------ 0

HLA-A*01:01:01:01 ATAATGTATGGCTGCGACGTGGGGCCGGACGGGCGCTTCCTCCGCGGGTACCGGCAGGAC 420

HLA-ABC ------------------------------------------------------------ 0

HLA-A*01:01:01:01 GCCTACGACGGCAAGGATTACATCGCCCTGAACGAGGACCTGCGCTCTTGGACCGCGGCG 480

HLA-ABC ------------------------------------------------------------ 0

HLA-A*01:01:01:01 GACATGGCAGCTCAGATCACCAAGCGCAAGTGGGAGGCGGTCCATGCGGCGGAGCAGCGG 540

HLA-ABC ------------------------------------------------------------ 0

HLA-A*01:01:01:01 AGAGTCTACCTGGAGGGCCGGTGCGTGGACGGGCTCCGCAGATACCTGGAGAACGGGAAG 600

HLA-ABC ------------------------------------------------------------ 0

HLA-A*01:01:01:01 GAGACGCTGCAGCGCACGGACCCCCCCAAGACACATATGACCCACCACCCCATCTCTGAC 660

HLA-ABC ------------------------------------------------------------ 0

HLA-A*01:01:01:01 CATGAGGCCACCCTGAGGTGCTGGGCCCTGGGCTTCTACCCTGCGGAGATCACACTGACC 720

HLA-ABC ------------------------------------------------------------ 0

HLA-A*01:01:01:01 TGGCAGCGGGATGGGGAGGACCAGACCCAGGACACGGAGCTCGTGGAGACCAGGCCTGCA 780

HLA-ABC ------------------------------------------------------------ 0

HLA-A*01:01:01:01 GGGGATGGAACCTTCCAGAAGTGGGCGGCTGTGGTGGTGCCTTCTGGAGAGGAGCAGAGA 840

HLA-ABC ------------------------------------------------------------ 0

HLA-A*01:01:01:01 TACACCTGCCATGTGCAGCATGAGGGTCTGCCCAAGCCCCTCACCCTGAGATGGGAGCTG 900

HLA-ABC ------------------------------------------------------------ 0

HLA-A*01:01:01:01 TCTTCCCAGCCCACCATCCCCATCGTGGGCATCATTGCTGGCCTGGTTCTCCTTGGAGCT 960

HLA-ABC ------------------------------------------------------------ 0

HLA-A*01:01:01:01 GTGATCACTGGAGCTGTGGTCGCTGCCGTGATGTGGAGGAGGAAGAGCTCAGATAGAAAA 1020

HLA-ABC ------------------------------------------------------------ 0

HLA-A*01:01:01:01 GGAGGGAGTTACACTCAGGCTGCAAGCAGTG**ACAGTGCCCAGGGCTCTGAT**GTGTCTCTC 1080

HLA-ABC -------------------------------**ACAGTGCCCAGGGCTCTGAT**--------- 20

********************

HLA-A*01:01:01:01 ACAGCTTGTAAAGTGTGA 1098

HLA-ABC ------------------ 20

**HLA:HLA00132 B*07:02:01:01 1089 bp**

**HLA-ABC gene-specific primer (amplicon length 1074 bp)**

HLA-B*07:02:01:01 ATGCTGGTCATGGCGCCCCGAACCGTCCTCCTGCTGCTCTCGGCGGCCCTGGCCCTGACC 60

HLA-ABC ------------------------------------------------------------ 0

HLA-B*07:02:01:01 GAGACCTGGGCCGGCTCCCACTCCATGAGGTATTTCTACACCTCCGTGTCCCGGCCCGGC 120

HLA-ABC ------------------------------------------------------------ 0

HLA-B*07:02:01:01 CGCGGGGAGCCCCGCTTCATCTCAGTGGGCTACGTGGACGACACCCAGTTCGTGAGGTTC 180

HLA-ABC ------------------------------------------------------------ 0

HLA-B*07:02:01:01 GACAGCGACGCCGCGAGTCCGAGAGAGGAGCCGCGGGCGCCGTGGATAGAGCAGGAGGGG 240

HLA-ABC ------------------------------------------------------------ 0

HLA-B*07:02:01:01 CCGGAGTATTGGGACCGGAACACACAGATCTACAAGGCCCAGGCACAGACTGACCGAGAG 300

HLA-ABC ------------------------------------------------------------ 0

HLA-B*07:02:01:01 AGCCTGCGGAACCTGCGCGGCTACTACAACCAGAGCGAGGCCGGGTCTCACACCCTCCAG 360

HLA-ABC ------------------------------------------------------------ 0

HLA-B*07:02:01:01 AGCATGTACGGCTGCGACGTGGGGCCGGACGGGCGCCTCCTCCGCGGGCATGACCAGTAC 420

HLA-ABC ------------------------------------------------------------ 0

HLA-B*07:02:01:01 GCCTACGACGGCAAGGATTACATCGCCCTGAACGAGGACCTGCGCTCCTGGACCGCCGCG 480

HLA-ABC ------------------------------------------------------------ 0

HLA-B*07:02:01:01 GACACGGCGGCTCAGATCACCCAGCGCAAGTGGGAGGCGGCCCGTGAGGCGGAGCAGCGG 540

HLA-ABC ------------------------------------------------------------ 0

HLA-B*07:02:01:01 AGAGCCTACCTGGAGGGCGAGTGCGTGGAGTGGCTCCGCAGATACCTGGAGAACGGGAAG 600

HLA-ABC ------------------------------------------------------------ 0

HLA-B*07:02:01:01 GACAAGCTGGAGCGCGCTGACCCCCCAAAGACACACGTGACCCACCACCCCATCTCTGAC 660

HLA-ABC ------------------------------------------------------------ 0

HLA-B*07:02:01:01 CATGAGGCCACCCTGAGGTGCTGGGCCCTGGGTTTCTACCCTGCGGAGATCACACTGACC 720

HLA-ABC ------------------------------------------------------------ 0

HLA-B*07:02:01:01 TGGCAGCGGGATGGCGAGGACCAAACTCAGGACACTGAGCTTGTGGAGACCAGACCAGCA 780

HLA-ABC ------------------------------------------------------------ 0

HLA-B*07:02:01:01 GGAGATAGAACCTTCCAGAAGTGGGCAGCTGTGGTGGTGCCTTCTGGAGAAGAGCAGAGA 840

HLA-ABC ------------------------------------------------------------ 0

HLA-B*07:02:01:01 TACACATGCCATGTACAGCATGAGGGGCTGCCGAAGCCCCTCACCCTGAGATGGGAGCCG 900

HLA-ABC ------------------------------------------------------------ 0

HLA-B*07:02:01:01 TCTTCCCAGTCCACCGTCCCCATCGTGGGCATTGTTGCTGGCCTGGCTGTCCTAGCAGTT 960

HLA-ABC ------------------------------------------------------------ 0

HLA-B*07:02:01:01 GTGGTCATCGGAGCTGTGGTCGCTGCTGTGATGTGTAGGAGGAAGAGTTCAGGTGGAAAA 1020

HLA-ABC ------------------------------------------------------------ 0

HLA-B*07:02:01:01 GGAGGGAGCTACTCTCAGGCTGCGTGCAGCG**ACAGTGCCCAGGGCTCTGAT**GTGTCTCTC 1080

HLA-ABC -------------------------------**ACAGTGCCCAGGGCTCTGAT**--------- 20

********************

HLA-B*07:02:01:01 ACAGCTTGA 1089

HLA-ABC --------- 20

**HLA:HLA00401 C*01:02:01:01 1101 bp**

**HLA-ABC gene-specific primer (amplicon length 1074 bp)**

HLA-C*01:02:01:01 ATGCGGGTCATGGCGCCCCGAACCCTCATCCTGCTGCTCTCGGGAGCCCTGGCCCTGACC 60

HLA-ABC ------------------------------------------------------------ 0

HLA-C*01:02:01:01 GAGACCTGGGCCTGCTCCCACTCCATGAAGTATTTCTTCACATCCGTGTCCCGGCCTGGC 120

HLA-ABC ------------------------------------------------------------ 0

HLA-C*01:02:01:01 CGCGGAGAGCCCCGCTTCATCTCAGTGGGCTACGTGGACGACACGCAGTTCGTGCGGTTC 180

HLA-ABC ------------------------------------------------------------ 0

HLA-C*01:02:01:01 GACAGCGACGCCGCGAGTCCGAGAGGGGAGCCGCGGGCGCCGTGGGTGGAGCAGGAGGGG 240

HLA-ABC ------------------------------------------------------------ 0

HLA-C*01:02:01:01 CCGGAGTATTGGGACCGGGAGACACAGAAGTACAAGCGCCAGGCACAGACTGACCGAGTG 300

HLA-ABC ------------------------------------------------------------ 0

HLA-C*01:02:01:01 AGCCTGCGGAACCTGCGCGGCTACTACAACCAGAGCGAGGCCGGGTCTCACACCCTCCAG 360

HLA-ABC ------------------------------------------------------------ 0

HLA-C*01:02:01:01 TGGATGTGTGGCTGCGACCTGGGGCCCGACGGGCGCCTCCTCCGCGGGTATGACCAGTAC 420

HLA-ABC ------------------------------------------------------------ 0

HLA-C*01:02:01:01 GCCTACGACGGCAAGGATTACATCGCCCTGAACGAGGACCTGCGCTCCTGGACCGCCGCG 480

HLA-ABC ------------------------------------------------------------ 0

HLA-C*01:02:01:01 GACACCGCGGCTCAGATCACCCAGCGCAAGTGGGAGGCGGCCCGTGAGGCGGAGCAGCGG 540

HLA-ABC ------------------------------------------------------------ 0

HLA-C*01:02:01:01 AGAGCCTACCTGGAGGGCACGTGCGTGGAGTGGCTCCGCAGATACCTGGAGAACGGGAAG 600

HLA-ABC ------------------------------------------------------------ 0

HLA-C*01:02:01:01 GAGACGCTGCAGCGCGCGGAACACCCAAAGACACACGTGACCCACCATCCCGTCTCTGAC 660

HLA-ABC ------------------------------------------------------------ 0

HLA-C*01:02:01:01 CATGAGGCCACCCTGAGGTGCTGGGCCCTGGGCTTCTACCCTGCGGAGATCACACTGACC 720

HLA-ABC ------------------------------------------------------------ 0

HLA-C*01:02:01:01 TGGCAGTGGGATGGGGAGGACCAAACTCAGGACACCGAGCTTGTGGAGACCAGGCCAGCA 780

HLA-ABC ------------------------------------------------------------ 0

HLA-C*01:02:01:01 GGAGATGGAACCTTCCAGAAGTGGGCAGCTGTGATGGTGCCTTCTGGAGAAGAGCAGAGA 840

HLA-ABC ------------------------------------------------------------ 0

HLA-C*01:02:01:01 TACACGTGCCATGTGCAGCACGAGGGGCTGCCGGAGCCCCTCACCCTGAGATGGGAGCCG 900

HLA-ABC ------------------------------------------------------------ 0

HLA-C*01:02:01:01 TCTTCCCAGCCCACCATCCCCATCGTGGGCATCGTTGCTGGCCTGGCTGTCCTGGCTGTC 960

HLA-ABC ------------------------------------------------------------ 0

HLA-C*01:02:01:01 CTAGCTGTCCTAGGAGCTGTGGTGGCTGTTGTGATGTGTAGGAGGAAGAGCTCAGGTGGA 1020

HLA-ABC ------------------------------------------------------------ 0

HLA-C*01:02:01:01 AAAGGAGGGAGCTGCTCTCAGGCTGCGTCCAGCA**ACAGTGCCCAGGGCTCTGAT**GAGTCT 1080

HLA-ABC ----------------------------------**ACAGTGCCCAGGGCTCTGAT**------ 20

********************

HLA-C*01:02:01:01 CTCATCGCTTGTAAAGCCTGA 1101

HLA-ABC --------------------- 20

**HLA:HLA00662 DRA*01:01:01:01 765 bp**

**HLA-DRA gene-specific primer (amplicon length 628 bp)**

HLA-DRA*01:01:01:01 ATGGCCATAAGTGGAGTCCCTGTGCTAGGATTTTTCATCATAGCTGTGCTGATGAGCGCT 60

HLA-DRA ------------------------------------------------------------ 0

HLA-DRA*01:01:01:01 CAGGAATCATGGGCTATCAAAGAAGAACATGTGATCATCCAGGCCGAGTTCTATCTGAAT 120

HLA-DRA ------------------------------------------------------------ 0

HLA-DRA*01:01:01:01 CCTGACCAATCAGGCGAGTTTATGTTTGACTTTGATGGTGATGAGATTTTCCATGTGGAT 180

HLA-DRA ------------------------------------------------------------ 0

HLA-DRA*01:01:01:01 ATGGCAAAGAAGGAGACGGTCTGGCGGCTTGAAGAATTTGGACGATTTGCCAGCTTTGAG 240

HLA-DRA ------------------------------------------------------------ 0

HLA-DRA*01:01:01:01 GCTCAAGGTGCATTGGCCAACATAGCTGTGGACAAAGCCAACCTGGAAATCATGACAAAG 300

HLA-DRA ------------------------------------------------------------ 0

HLA-DRA*01:01:01:01 CGCTCCAACTATACTCCGATCACCAATGTACCTCCAGAGGTAACTGTGCTCACGAACAGC 360

HLA-DRA ------------------------------------------------------------ 0

HLA-DRA*01:01:01:01 CCTGTGGAACTGAGAGAGCCCAACGTCCTCATCTGTTTCATCGACAAGTTCACCCCACCA 420

HLA-DRA ------------------------------------------------------------ 0

HLA-DRA*01:01:01:01 GTGGTCAATGTCACGTGGCTTCGAAATGGAAAACCTGTCACCACAGGAGTGTCAGAGACA 480

HLA-DRA ------------------------------------------------------------ 0

HLA-DRA*01:01:01:01 GTCTTCCTGCCCAGGGAAGACCACCTTTTCCGCAAGTTCCACTATCTCCCCTTCCTGCCC 540

HLA-DRA ------------------------------------------------------------ 0

HLA-DRA*01:01:01:01 TCAACTGAGGACGTTTACGACTGCAGGGTGGAGCACTGGGGCTTGGATGAGCCTCTTCTC 600

HLA-DRA ------------------------------------------------------------ 0

HLA-DRA*01:01:01:01 AAG**CACTGGGAGTTTGATGCTCCAAGCC**CTCTCCCAGAGACTACAGAGAACGTGGTGTGT 660

HLA-DRA ---**CACTGGGAGTTTGATGCTCCAAGCC**-------------------------------- 25

*************************

HLA-DRA*01:01:01:01 GCCCTGGGCCTGACTGTGGGTCTGGTGGGCATCATTATTGGGACCATCTTCATCATCAAG 720

HLA-DRA ------------------------------------------------------------ 25

HLA-DRA*01:01:01:01 GGAGTGCGCAAAAGCAATGCAGCAGAACGCAGGGGGCCTCTGTAA 765

HLA-DRA --------------------------------------------- 25

**HLA:HLA00664 DRB1*01:01:01:01 801 bp**

**HLA-DRB gene-specific primer (amplicon length 694 bp)**

HLA-DRB1*01:01:01:01 ATGGTGTGTCTGAAGCTCCCTGGAGGCTCCTGCATGACAGCGCTGACAGTGACACTGATG 60

HLA-DRB ------------------------------------------------------------ 0

HLA-DRB1*01:01:01:01 GTGCTGAGCTCCCCACTGGCTTTGGCTGGGGACACCCGACCACGTTTCTTGTGGCAGCTT 120

HLA-DRB ------------------------------------------------------------ 0

HLA-DRB1*01:01:01:01 AAGTTTGAATGTCATTTCTTCAATGGGACGGAGCGGGTGCGGTTGCTGGAAAGATGCATC 180

HLA-DRB ------------------------------------------------------------ 0

HLA-DRB1*01:01:01:01 TATAACCAAGAGGAGTCCGTGCGCTTCGACAGCGACGTGGGGGAGTACCGGGCGGTGACG 240

HLA-DRB ------------------------------------------------------------ 0

HLA-DRB1*01:01:01:01 GAGCTGGGGCGGCCTGATGCCGAGTACTGGAACAGCCAGAAGGACCTCCTGGAGCAGAGG 300

HLA-DRB ------------------------------------------------------------ 0

HLA-DRB1*01:01:01:01 CGGGCCGCGGTGGACACCTACTGCAGACACAACTACGGGGTTGGTGAGAGCTTCACAGTG 360

HLA-DRB ------------------------------------------------------------ 0

HLA-DRB1*01:01:01:01 CAGCGGCGAGTTGAGCCTAAGGTGACTGTGTATCCTTCAAAGACCCAGCCCCTGCAGCAC 420

HLA-DRB ------------------------------------------------------------ 0

HLA-DRB1*01:01:01:01 CACAACCTCCTGGTCTGCTCTGTGAGTGGTTTCTATCCAGGCAGCATTGAAGTCAGGTGG 480

HLA-DRB ------------------------------------------------------------ 0

HLA-DRB1*01:01:01:01 TTCCGGAACGGCCAGGAAGAGAAGGCTGGGGTGGTGTCCACAGGCCTGATCCAGAATGGA 540

HLA-DRB ------------------------------------------------------------ 0

HLA-DRB1*01:01:01:01 GATTGGACCTTCCAGACCCTGGTGATGCTGGAAACAGTTCCTCGGAGTGGAGAGGTTTAC 600

HLA-DRB ------------------------------------------------------------ 0

HLA-DRB1*01:01:01:01 ACCTGCCAAGTGGAGCACCCAAGTGTGACGAGCCCTCTCACAGTGGAATGGAGAGCACGG 660

HLA-DRB ------------------------------------------------------------ 0

HLA-DRB1*01:01:01:01 TCTGAATC**TGCACAGAGCAAGATGCTGAGTGGAG**TCGGGGGCTTCGTGCTGGGCCTGCTC 720

HLA-DRB --------**TGCACAGAGCAAGATGCTGAGTGGAG**-------------------------- 26

**************************

HLA-DRB1*01:01:01:01 TTCCTTGGGGCCGGGCTGTTCATCTACTTCAGGAATCAGAAAGGACACTCTGGACTTCAG 780

HLA-DRB ------------------------------------------------------------ 26

HLA-DRB1*01:01:01:01 CCAACAGGATTCCTGAGCTGA 801

HLA-DRB --------------------- 26

**HLA:HLA00887 DRB3*01:01:02:01 801 bp**

**HLA-DRB gene-specific primer (amplicon length 694 bp)**

DRB3*01:01:02:01 ATGGTGTGTCTGAAGCTCCCTGGAGGCTCCAGCTTGGCAGCGTTGACAGTGACACTGATG 60

HLA-DRB ------------------------------------------------------------ 0

DRB3*01:01:02:01 GTGCTGAGCTCCCGACTGGCTTTCGCTGGGGACACCCGACCACGTTTCTTGGAGCTGCGT 120

HLA-DRB ------------------------------------------------------------ 0

DRB3*01:01:02:01 AAGTCTGAGTGTCATTTCTTCAATGGGACGGAGCGGGTGCGGTACCTGGACAGATACTTC 180

HLA-DRB ------------------------------------------------------------ 0

DRB3*01:01:02:01 CATAACCAGGAGGAGTTCCTGCGCTTCGACAGCGACGTGGGGGAGTACCGGGCGGTGACG 240

HLA-DRB ------------------------------------------------------------ 0

DRB3*01:01:02:01 GAGCTGGGGCGGCCTGTCGCCGAGTCCTGGAACAGCCAGAAGGACCTCCTGGAGCAGAAG 300

HLA-DRB ------------------------------------------------------------ 0

DRB3*01:01:02:01 CGGGGCCGGGTGGACAATTACTGCAGACACAACTACGGGGTTGGTGAGAGCTTCACAGTG 360

HLA-DRB ------------------------------------------------------------ 0

DRB3*01:01:02:01 CAGCGGCGAGTCCATCCTCAGGTGACTGTGTATCCTGCAAAGACCCAGCCCCTGCAGCAC 420

HLA-DRB ------------------------------------------------------------ 0

DRB3*01:01:02:01 CACAACCTCCTGGTCTGCTCTGTGAGTGGTTTCTATCCAGGCAGCATTGAAGTCAGGTGG 480

HLA-DRB ------------------------------------------------------------ 0

DRB3*01:01:02:01 TTCCGGAACGGCCAGGAAGAGAAGGCTGGGGTGGTGTCCACGGGCCTGATCCAGAATGGA 540

HLA-DRB ------------------------------------------------------------ 0

DRB3*01:01:02:01 GACTGGACCTTCCAGACCCTGGTGATGCTAGAAACAGTTCCTCGGAGTGGAGAGGTTTAC 600

HLA-DRB ------------------------------------------------------------ 0

DRB3*01:01:02:01 ACTTGCCAAGTGGAGCACCCAAGCGTAACGAGCGCTCTCACAGTGGAATGGAGAGCACGG 660

HLA-DRB ------------------------------------------------------------ 0

DRB3*01:01:02:01 TCTGAATC**TGCACAGAGCAAGATGCTGAGTGGAG**TCGGGGGCTTTGTGCTGGGCCTGCTC 720

HLA-DRB --------**TGCACAGAGCAAGATGCTGAGTGGAG**-------------------------- 26

**************************

DRB3*01:01:02:01 TTCCTTGGGGCCGGGCTGTTCATCTACTTCAGGAATCAGAAAGGACACTCTGGACTTCAG 780

HLA-DRB ------------------------------------------------------------ 26

DRB3*01:01:02:01 CCAACAGGATTCCTGAGCTGA 801

HLA-DRB --------------------- 26

**HLA:HLA00908 DRB4*01:03:01:01 801 bp**

**HLA-DRB gene-specific primer (amplicon length 694 bp)**

DRB4*01:03:01:01 ATGGTGTGTCTGAAGCTCCCTGGAGGCTCCTGTATGGCAGCGCTGACAGTGACATTGACG 60

HLA-DRB ------------------------------------------------------------ 0

DRB4*01:03:01:01 GTGCTGAGCTCCCCACTGGCTTTGGCTGGGGACACCCAACCACGTTTCTTGGAGCAGGCT 120

HLA-DRB ------------------------------------------------------------ 0

DRB4*01:03:01:01 AAGTGTGAGTGTCATTTCCTCAATGGGACGGAGCGAGTGTGGAACCTGATCAGATACATC 180

HLA-DRB ------------------------------------------------------------ 0

DRB4*01:03:01:01 TATAACCAAGAGGAGTACGCGCGCTACAACAGTGACCTGGGGGAGTACCAGGCGGTGACG 240

HLA-DRB ------------------------------------------------------------ 0

DRB4*01:03:01:01 GAGCTGGGGCGGCCTGACGCTGAGTACTGGAACAGCCAGAAGGACCTCCTGGAGCGGAGG 300

HLA-DRB ------------------------------------------------------------ 0

DRB4*01:03:01:01 CGGGCCGAGGTGGACACCTACTGCAGATACAACTACGGGGTTGTGGAGAGCTTCACAGTG 360

HLA-DRB ------------------------------------------------------------ 0

DRB4*01:03:01:01 CAGCGGCGAGTCCAACCTAAGGTGACTGTGTATCCTTCAAAGACCCAGCCCCTGCAGCAC 420

HLA-DRB ------------------------------------------------------------ 0

DRB4*01:03:01:01 CACAACCTCCTGGTCTGCTCTGTGAATGGTTTCTATCCAGGCAGCATTGAAGTCAGGTGG 480

HLA-DRB ------------------------------------------------------------ 0

DRB4*01:03:01:01 TTCCGGAACGGCCAGGAAGAGAAGGCTGGGGTGGTGTCCACAGGCCTGATCCAGAATGGA 540

HLA-DRB ------------------------------------------------------------ 0

DRB4*01:03:01:01 GACTGGACCTTCCAGACCCTGGTGATGCTGGAAACAGTTCCTCGGAGTGGAGAGGTTTAC 600

HLA-DRB ------------------------------------------------------------ 0

DRB4*01:03:01:01 ACCTGCCAAGTGGAGCATCCAAGCATGATGAGCCCTCTCACGGTGCAATGGAGTGCACGG 660

HLA-DRB ------------------------------------------------------------ 0

DRB4*01:03:01:01 TCTGAATC**TGCACAGAGCAAGATGCTGAGTGGAG**TCGGGGGCTTTGTGCTGGGCCTGCTC 720

HLA-DRB --------**TGCACAGAGCAAGATGCTGAGTGGAG**-------------------------- 26

**************************

DRB4*01:03:01:01 TTCCTTGGGACAGGGCTGTTCATCTACTTCAGGAATCAGAAAGGACACTCTGGACTTCAG 780

HLA-DRB ------------------------------------------------------------ 26

DRB4*01:03:01:01 CCAACAGGACTCTTGAGCTGA 801

HLA-DRB --------------------- 26

**HLA:HLA00915 DRB5*01:01:01:01 801 bp**

**HLA-DRB gene-specific primer (amplicon length 694 bp)**

HLA-DRB5*01:01:01:01 ATGGTGTGTCTGAAGCTCCCTGGAGGTTCCTACATGGCAAAGCTGACAGTGACACTGATG 60

HLA-DRB ------------------------------------------------------------ 0

HLA-DRB5*01:01:01:01 GTGCTGAGCTCCCCACTGGCTTTGGCTGGGGACACCCGACCACGTTTCTTGCAGCAGGAT 120

HLA-DRB ------------------------------------------------------------ 0

HLA-DRB5*01:01:01:01 AAGTATGAGTGTCATTTCTTCAACGGGACGGAGCGGGTGCGGTTCCTGCACAGAGACATC 180

HLA-DRB ------------------------------------------------------------ 0

HLA-DRB5*01:01:01:01 TATAACCAAGAGGAGGACTTGCGCTTCGACAGCGACGTGGGGGAGTACCGGGCGGTGACG 240

HLA-DRB ------------------------------------------------------------ 0

HLA-DRB5*01:01:01:01 GAGCTGGGGCGGCCTGACGCTGAGTACTGGAACAGCCAGAAGGACTTCCTGGAAGACAGG 300

HLA-DRB ------------------------------------------------------------ 0

HLA-DRB5*01:01:01:01 CGCGCCGCGGTGGACACCTACTGCAGACACAACTACGGGGTTGGTGAGAGCTTCACAGTG 360

HLA-DRB ------------------------------------------------------------ 0

HLA-DRB5*01:01:01:01 CAGCGGCGAGTTGAGCCTAAGGTGACTGTGTATCCTGCAAGGACCCAGACCCTGCAGCAC 420

HLA-DRB ------------------------------------------------------------ 0

HLA-DRB5*01:01:01:01 CACAACCTCCTGGTCTGCTCTGTGAATGGTTTCTATCCAGGCAGCATTGAAGTCAGGTGG 480

HLA-DRB ------------------------------------------------------------ 0

HLA-DRB5*01:01:01:01 TTCCGGAACAGCCAGGAAGAGAAGGCTGGGGTGGTGTCCACAGGCCTGATTCAGAATGGA 540

HLA-DRB ------------------------------------------------------------ 0

HLA-DRB5*01:01:01:01 GACTGGACCTTCCAGACCCTGGTGATGCTGGAAACAGTTCCTCGAAGTGGAGAGGTTTAC 600

HLA-DRB ------------------------------------------------------------ 0

HLA-DRB5*01:01:01:01 ACCTGCCAAGTGGAGCACCCAAGCGTGACGAGCCCTCTCACAGTGGAATGGAGAGCACAG 660

HLA-DRB ------------------------------------------------------------ 0

HLA-DRB5*01:01:01:01 TCTGAATC**TGCACAGAGCAAGATGCTGAGTGGAG**TCGGGGGCTTTGTGCTGGGCCTGCTC 720

HLA-DRB --------**TGCACAGAGCAAGATGCTGAGTGGAG**-------------------------- 26

**************************

HLA-DRB5*01:01:01:01 TTCCTTGGGGCCGGGCTATTCATCTACTTCAAGAATCAGAAAGGGCACTCTGGACTTCAC 780

HLA-DRB ------------------------------------------------------------ 26

HLA-DRB5*01:01:01:01 CCAACAGGACTCGTGAGCTGA 801

HLA-DRB --------------------- 26

**HLA:HLA00499 DPA1*01:03:01:01 783 bp**

**HLA-DPA1 gene-specific primer (amplicon length 748 bp)**

HLA-DPA1*01:03:01:01 ATGCGCCCTGAAGACAGAATGTTCCATATCAGAGCTGTGATCTTGAGAGCCCTCTCCTTG 60

HLA-DPA1 ------------------------------------------------------------ 0

HLA-DPA1*01:03:01:01 GCTTTCCTGCTGAGTCTCCGAGGAGCTGGGGCCATCAAGGCGGACCATGTGTCAACTTAT 120

HLA-DPA1 ------------------------------------------------------------ 0

HLA-DPA1*01:03:01:01 GCCGCGTTTGTACAGACGCATAGACCAACAGGGGAGTTTATGTTTGAATTTGATGAAGAT 180

HLA-DPA1 ------------------------------------------------------------ 0

HLA-DPA1*01:03:01:01 GAGATGTTCTATGTGGATCTGGACAAGAAGGAGACCGTCTGGCATCTGGAGGAGTTTGGC 240

HLA-DPA1 ------------------------------------------------------------ 0

HLA-DPA1*01:03:01:01 CAAGCCTTTTCCTTTGAGGCTCAGGGCGGGCTGGCTAACATTGCTATATTGAACAACAAC 300

HLA-DPA1 ------------------------------------------------------------ 0

HLA-DPA1*01:03:01:01 TTGAATACCTTGATCCAGCGTTCCAACCACACTCAGGCCACCAACGATCCCCCTGAGGTG 360

HLA-DPA1 ------------------------------------------------------------ 0

HLA-DPA1*01:03:01:01 ACCGTGTTTCCCAAGGAGCCTGTGGAGCTGGGCCAGCCCAACACCCTCATCTGCCACATT 420

HLA-DPA1 ------------------------------------------------------------ 0

HLA-DPA1*01:03:01:01 GACAAGTTCTTCCCACCAGTGCTCAACGTCACGTGGCTGTGCAACGGGGAGCTGGTCACT 480

HLA-DPA1 ------------------------------------------------------------ 0

HLA-DPA1*01:03:01:01 GAGGGTGTCGCTGAGAGCCTCTTCCTGCCCAGAACAGATTACAGCTTCCACAAGTTCCAT 540

HLA-DPA1 ------------------------------------------------------------ 0

HLA-DPA1*01:03:01:01 TACCTGACCTTTGTGCCCTCAGCAGAGGACTTCTATGACTGCAGGGTGGAGCACTGGGGC 600

HLA-DPA1 ------------------------------------------------------------ 0

HLA-DPA1*01:03:01:01 TTGGACCAGCCGCTCCTCAAGCACTGGGAGGCCCAAGAGCCAATCCAGATGCCTGAGACA 660

HLA-DPA1 ------------------------------------------------------------ 0

HLA-DPA1*01:03:01:01 ACGGAGACTGTGCTCTGTGCCCTGGGCCTGGTGCTGGGCCTAGTCGGCATCATCGTGGGC 720

HLA-DPA1 ------------------------------------------------------------ 0

HLA-DPA1*01:03:01:01 **ACCGTCCTCATCATAAAGTCTCTGCGTT**CTGGCCATGACCCCCGGGCCCAGGGGACCCTG 780

HLA-DPA1  **ACCGTCCTCATCATAAAGTCTCTGCGTT**-------------------------------- 28

****************************

HLA-DPA1*01:03:01:01 TGA 783

HLA-DPA1 --- 28

**HLA:HLA00517 DPB1*02:01:02:01 777 bp**

**HLA-DPB1 gene-specific primer (amplicon length 628 bp)**

HLA-DPB1*02:01:02:01 ATGATGGTTCTGCAGGTTTCTGCGGCCCCCCGGACAGTGGCTCTGACGGCGTTACTGATG 60

HLA-DPB1 ------------------------------------------------------------ 0

HLA-DPB1*02:01:02:01 GTGCTGCTCACATCTGTGGTCCAGGGCAGGGCCACTCCAGAGAATTACCTTTTCCAGGGA 120

HLA-DPB1 ------------------------------------------------------------ 0

HLA-DPB1*02:01:02:01 CGGCAGGAATGCTACGCGTTTAATGGGACACAGCGCTTCCTGGAGAGATACATCTACAAC 180

HLA-DPB1 ------------------------------------------------------------ 0

HLA-DPB1*02:01:02:01 CGGGAGGAGTTCGTGCGCTTCGACAGCGACGTGGGGGAGTTCCGGGCGGTGACGGAGCTG 240

HLA-DPB1 ------------------------------------------------------------ 0

HLA-DPB1*02:01:02:01 GGGCGGCCTGATGAGGAGTACTGGAACAGCCAGAAGGACATCCTGGAGGAGGAGCGGGCA 300

HLA-DPB1 ------------------------------------------------------------ 0

HLA-DPB1*02:01:02:01 GTGCCGGACAGGATGTGCAGACACAACTACGAGCTGGGCGGGCCCATGACCCTGCAGCGC 360

HLA-DPB1 ------------------------------------------------------------ 0

HLA-DPB1*02:01:02:01 CGAGTCCAGCCTAGGGTGAATGTTTCCCCCTCCAAGAAGGGGCCCTTGCAGCACCACAAC 420

HLA-DPB1 ------------------------------------------------------------ 0

HLA-DPB1*02:01:02:01 CTGCTTGTCTGCCACGTGACGGATTTCTACCCAGGCAGCATTCAAGTCCGATGGTTCCTG 480

HLA-DPB1 ------------------------------------------------------------ 0

HLA-DPB1*02:01:02:01 AATGGACAGGAGGAAACAGCTGGGGTCGTGTCCACCAACCTGATCCGTAATGGAGACTGG 540

HLA-DPB1 ------------------------------------------------------------ 0

HLA-DPB1*02:01:02:01 ACCTTCCAGATCCTGGTGATGCTGGAAATGACCCCCCAGCAGGGAGATGTCTACACCTGC 600

HLA-DPB1 ------------------------------------------------------------ 0

HLA-DPB1*02:01:02:01 CAA**GTGGAGCACACCAGCCTGGATAGTC**CTGTCACCGTGGAGTGGAAGGCACAGTCTGAT 660

HLA-DPB1 ---**GTGGAGCACACCAGCCTGGATAGTC**-------------------------------- 25

*************************

HLA-DPB1*02:01:02:01 TCTGCCCGGAGTAAGACATTGACGGGAGCTGGGGGCTTCGTGCTGGGGCTCATCATCTGT 720

HLA-DPB1 ------------------------------------------------------------ 25

HLA-DPB1*02:01:02:01 GGAGTGGGCATCTTCATGCACAGGAGGAGCAAGAAAGTTCAACGAGGATCTGCATAA 777

HLA-DPB1 --------------------------------------------------------- 25

**HLA:HLA00601 DQA1*01:01:01:01 768 bp**

**HLA-DQA1 gene-specific primer (amplicon length 757 bp)**

HLA-DQA1*01:01:01:01 ATGATCCTAAACAAAGCTCTGCTGCTGGGGGCCCTCGCTCTGACCACCGTGATGAGCCCC 60

HLA-DQA1 ------------------------------------------------------------ 0

HLA-DQA1*01:01:01:01 TGTGGAGGTGAAGACATTGTGGCTGACCACGTTGCCTCTTGTGGTGTAAACTTGTACCAG 120

HLA-DQA1 ------------------------------------------------------------ 0

HLA-DQA1*01:01:01:01 TTTTACGGTCCCTCTGGCCAGTACACCCATGAATTTGATGGAGATGAGGAGTTCTACGTG 180

HLA-DQA1 ------------------------------------------------------------ 0

HLA-DQA1*01:01:01:01 GACCTGGAGAGGAAGGAGACTGCCTGGCGGTGGCCTGAGTTCAGCAAATTTGGAGGTTTT 240

HLA-DQA1 ------------------------------------------------------------ 0

HLA-DQA1*01:01:01:01 GACCCGCAGGGTGCACTGAGAAACATGGCTGTGGCAAAACACAACTTGAACATCATGATT 300

HLA-DQA1 ------------------------------------------------------------ 0

HLA-DQA1*01:01:01:01 AAACGCTACAACTCTACCGCTGCTACCAATGAGGTTCCTGAGGTCACAGTGTTTTCCAAG 360

HLA-DQA1 ------------------------------------------------------------ 0

HLA-DQA1*01:01:01:01 TCTCCCGTGACACTGGGTCAGCCCAACACCCTCATTTGTCTTGTGGACAACATCTTTCCT 420

HLA-DQA1 ------------------------------------------------------------ 0

HLA-DQA1*01:01:01:01 CCTGTGGTCAACATCACATGGCTGAGCAATGGGCAGTCAGTCACAGAAGGTGTTTCTGAG 480

HLA-DQA1 ------------------------------------------------------------ 0

HLA-DQA1*01:01:01:01 ACCAGCTTCCTCTCCAAGAGTGATCATTCCTTCTTCAAGATCAGTTACCTCACCTTCCTC 540

HLA-DQA1 ------------------------------------------------------------ 0

HLA-DQA1*01:01:01:01 CCTTCTGCTGATGAGATTTATGACTGCAAGGTGGAGCACTGGGGCCTGGACCAGCCTCTT 600

HLA-DQA1 ------------------------------------------------------------ 0

HLA-DQA1*01:01:01:01 CTGAAACACTGGGAGCCTGAGATTCCAGCCCCTATGTCAGAGCTCACAGAGACTGTGGTC 660

HLA-DQA1 ------------------------------------------------------------ 0

HLA-DQA1*01:01:01:01 TGCGCCCTGGGGTTGTCTGTGGGCCTCGTGGGCATTGTGGTGGGCACTGTCTTCATCATC 720

HLA-DQA1 ------------------------------------------------------------ 0

HLA-DQA1*01:01:01:01 CAAGGCCTG**CGTTCAGTTGGTGCTTCCAGACACCAAG**GGCCATTGTGA 768

HLA-DQA1 ---------**CGTTCAGTTGGTGCTTCCAGACACCAAG**----------- 28

****************************

**HLA:HLA00622 DQB1*02:01:01:01 786 bp**

**HLA-DQB1 gene-specific primer (amplicon length 626 bp)**

HLA-DQB1*02:01:01:01 ATGTCTTGGAAAAAGGCTTTGCGGATCCCCGGAGGCCTTCGGGCAGCAACTGTGACCTTG 60

HLA-DQB1 ------------------------------------------------------------ 0

HLA-DQB1*02:01:01:01 ATGCTGTCGATGCTGAGCACCCCAGTGGCTGAGGGCAGAGACTCTCCCGAGGATTTCGTG 120

HLA-DQB1 ------------------------------------------------------------ 0

HLA-DQB1*02:01:01:01 TACCAGTTTAAGGGCATGTGCTACTTCACCAACGGGACAGAGCGCGTGCGTCTTGTGAGC 180

HLA-DQB1 ------------------------------------------------------------ 0

HLA-DQB1*02:01:01:01 AGAAGCATCTATAACCGAGAAGAGATCGTGCGCTTCGACAGCGACGTGGGGGAGTTCCGG 240

HLA-DQB1 ------------------------------------------------------------ 0

HLA-DQB1*02:01:01:01 GCGGTGACGCTGCTGGGGCTGCCTGCCGCCGAGTACTGGAACAGCCAGAAGGACATCCTG 300

HLA-DQB1 ------------------------------------------------------------ 0

HLA-DQB1*02:01:01:01 GAGAGGAAACGGGCGGCGGTGGACAGGGTGTGCAGACACAACTACCAGTTGGAGCTCCGC 360

HLA-DQB1 ------------------------------------------------------------ 0

HLA-DQB1*02:01:01:01 ACGACCTTGCAGCGGCGAGTGGAGCCCACAGTGACCATCTCCCCATCCAGGACAGAGGCC 420

HLA-DQB1 ------------------------------------------------------------ 0

HLA-DQB1*02:01:01:01 CTCAACCACCACAACCTGCTGGTCTGCTCGGTGACAGATTTCTATCCAGCCCAGATCAAA 480

HLA-DQB1 ------------------------------------------------------------ 0

HLA-DQB1*02:01:01:01 GTCCGGTGGTTTCGGAATGACCAGGAGGAGACAGCTGGCGTTGTGTCCACCCCCCTTATT 540

HLA-DQB1 ------------------------------------------------------------ 0

HLA-DQB1*02:01:01:01 AGGAATGGTGACTGGACCTTCCAGATCCTGGTGATGCTGGAAATGACTCCCCAGCGTGGA 600

HLA-DQB1 ------------------------------------------------------------ 0

HLA-DQB1*02:01:01:01 GAC**GTCTACACCTGCCACGTGGAGCA**CCCCAGCCTCCAGAGCCCCATCACCGTGGAGTGG 660

HLA-DQB1 ---**GTCTACACCTGCCACGTGGAGCA**---------------------------------- 23

***********************

HLA-DQB1*02:01:01:01 CGGGCTCAATCTGAATCTGCCCAGAGCAAGATGCTGAGTGGCATTGGAGGCTTCGTGCTG 720

HLA-DQB1 ------------------------------------------------------------ 23

HLA-DQB1*02:01:01:01 GGGCTGATCTTCCTCGGGCTGGGCCTTATCATCCATCACAGGAGTCAGAAAGGGCTCCTG 780

HLA-DQB1 ------------------------------------------------------------ 23

HLA-DQB1*02:01:01:01 CACTGA 786

HLA-DQB1 ------ 23

**Supplementary Figure S1. Binding sites of HLA gene-specific primers**. Clustal Omega (1.2.4) multiple sequence alignment showing the binding sites and amplicon lengths of seven HLA gene-specific primers (HLA-ABC, HLA-DRA, HLA-DRB, HLA-DPA1, HLA-DPB1, HLA-DQA1, and HLA-DQB1) for 12 HLA genes. HLA allele sequences were obtained from https://www.ebi.ac.uk/ipd/imgt/hla/.
