# Supplementary material for: Targeted RNA-Based Oxford Nanopore Sequencing for Typing 12 Classical HLA Genes
Source: Front Genet. 2021 Mar 4;12:635601. doi: 10.3389/fgene.2021.635601 (PMC7982845; doi:10.3389/fgene.2021.635601)
Supplement: Supplementary Figure 2 — Sequencing coverage graph for class I and class II in the SeqPilot interphase. [file Data_Sheet_2.docx]

**Supplementary Figure S2**

**A**

**Supplementary Figure S2. Sequencing coverage graph for class I and class II in the SeqPilot interphase.** Examples of the sequencing depths in the exon 1 and 2 in class I (A) and class II (B). Red boxes indicate the area of exons 1 and 2.


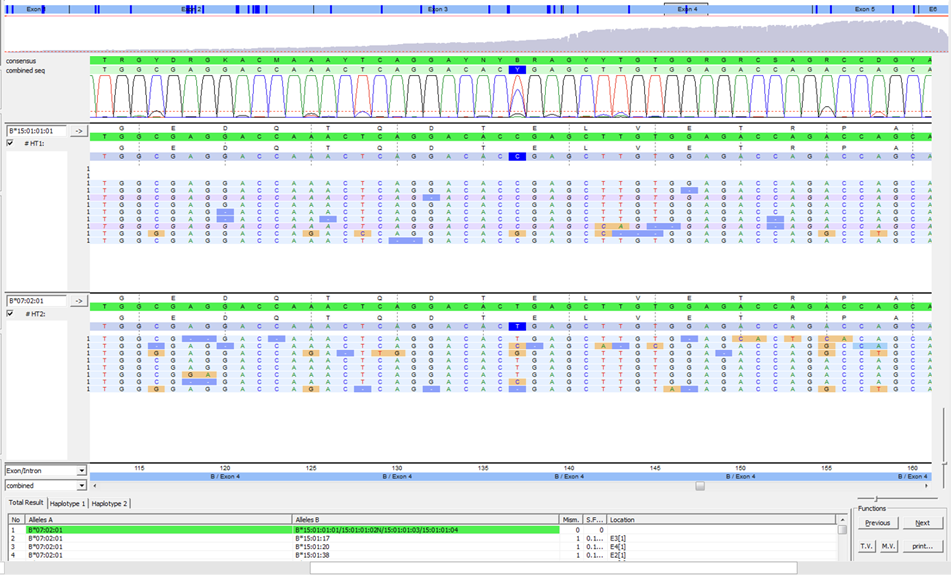


**B**
