# Supplementary material for: Targeted RNA-Based Oxford Nanopore Sequencing for Typing 12 Classical HLA Genes
Source: Front Genet. 2021 Mar 4;12:635601. doi: 10.3389/fgene.2021.635601 (PMC7982845; doi:10.3389/fgene.2021.635601)
Supplement: Supplementary Table 1 — List of primer sequences. [file Table_1.docx]

| Supplementary table S1. List of primer sequences. | |  |
| --- | --- | --- |
|  | |  |
|  | |  |
| Primer | **Primer sequence** | |
| STRT-V3-T30-VN oligo | 5’-biotin-TTAAGCAGTGGTATCAACGCAGAGTCGAC30VN-3’ | |
| RNA-TSO 10bp UMI | 5’-biotin-CAGUGGUAUCAACGCAGAGUNNNNNNNNNNrGrGrG-3’ | |
| ImSTRT-TSO-PCR | 5-’CAGTGGTATCAACGCAGAGT-3’ | |
|  |  | |
| Gene-specific primers |  | |
| TSO-specific universal forward | 5’-TTTCTGTTGGTGCTGATATTGCCAGTGGTATCAACGCAGAGT-3’ | |
| HLA-ABC-specific universal reverse | 5’- ACTTGCCTGTCGCTCTATCTTCATCAGAGCCCTGGGCACTGT-3’ | |
| DRA-specific universal reverse | 5’-ACTTGCCTGTCGCTCTATCTTCGGCTTGGAGCATCAAACTCCCAGTG-3’ | |
| DRB-specific universal reverse | 5’-ACTTGCCTGTCGCTCTATCTTCCTCCACTCAGCATCTTGCTCTGTGCA-3’ | |
| DPA1-specific universal reverse | 5’-ACTTGCCTGTCGCTCTATCTTCAACGCAGAGACTTTATGATGAGGACGGT-3’ | |
| DPB1-specific universal reverse | 5’-ACTTGCCTGTCGCTCTATCTTCGACTATCCAGGCTGGTGTGCTCCAC-3’ | |
| DQA1-specific universal reverse | 5’-ACTTGCCTGTCGCTCTATCTTCCTTGGTGTCTGGAAGCACCAACTGAACG-3’ | |
| DQB1-specific universal reverse | 5’-ACTTGCCTGTCGCTCTATCTTCTGCTCCACGTGGCAGGTGTAGAC-3’ | |

|  |  |
| --- | --- |
| In STRT-V3-T30-VN oligo, the recognition site of restriction enzyme SalI is underlined. In gene-specific primers, TSO-specific and HLA gene-specific parts of the primers are underlined. | |
