# Supplementary material for: Targeted RNA-Based Oxford Nanopore Sequencing for Typing 12 Classical HLA Genes
Source: Front Genet. 2021 Mar 4;12:635601. doi: 10.3389/fgene.2021.635601 (PMC7982845; doi:10.3389/fgene.2021.635601)
Supplement: Supplementary Table 2 — Alleles of 12 classical HLA genes of 50 individuals. [file Table_2.docx]

**Supplementary table S2. Alleles of 12 classical HLA genes of 50 individuals.**

| **HLA class I** |  |  | **HLA class II** |  |  |
| --- | --- | --- | --- | --- | --- |
| **HLA-A alleles** | **HLA-B alleles** | **HLA-C alleles** | **HLA-DRB3 alleles** | **HLA-DRB4 alleles** | **HLA-DRB5 alleles** |
| A*01:01 | B*07:02 | C*01:02 | DRB3*01:01 | DRB4*01:03 | DRB5*01:01 |
| A*02:01 | B*08:01 | C*02:02 | DRB3*02:02 |  | DRB5*02:02 |
| A*03:01 | B*13:02 | C*03:03 | DRB3*03:01 |  |  |
| A*03:03 | B*14:02 | C*03:04 |  |  |  |
| A*11:01 | B*15:01 | C*04:01 |  |  |  |
| A*24:02 | B*18:01 | C*05:01 |  |  |  |
| A*25:01 | B*27:02 | C*06:02 |  |  |  |
| A*26:01 | B*35:01 | C*07:01 |  |  |  |
| A*29:01 | B*35:03 | C*07:02 |  |  |  |
| A*29:02 | B*35:05 | C*07:04 |  |  |  |
| A*31:01 | B*35:08 | C*08:02 |  |  |  |
| A*32:01 | B*37:01 | C*12:03 |  |  |  |
| A*33:01 | B*39:01 | C*15:02 |  |  |  |
| A*68:01 | B*39:06 | C*17:01 |  |  |  |
|  | B*40:01 |  |  |  |  |
|  | B*41:01 |  |  |  |  |
|  | B*44:02 |  |  |  |  |
|  | B*44:27 |  |  |  |  |
|  | B*47:01 |  |  |  |  |
|  | B*49:01 |  |  |  |  |
|  | B*51:01 |  |  |  |  |
|  | B*55:01 |  |  |  |  |
|  | B*56:01 |  |  |  |  |
|  | B*57:01 |  |  |  |  |
|  |  |  |  |  |  |
|  |  |  |  |  |  |
|  |  |  |  |  |  |
| **HLA class II** |  |  |  |  |  |
| **HLA-DRA alleles*** | **HLA-DRB1 alleles** | **HLA-DPA1 alleles** | **HLA-DPB1 alleles** | **HLA-DQA1 alleles** | **HLA-DQB1 alleles** |
| DRA*01:01 | DRB1*01:01 | DPA1*01:03 | DPB1*01:01 | DQA1*01:01 | DQB1*02:01 |
| DRA*01:02 | DRB1*01:02 | DPA1*01:04 | DPB1*02:01 | DQA1*01:02 | DQB1*02:02 |
|  | DRB1*03:01 | DPA1*02:01 | DPB1*03:01 | DQA1*01:03 | DQB1*03:01 |
|  | DRB1*04:01 | DPA1*02:02 | DPB1*04:01 | DQA1*01:04 | DQB1*03:03 |
|  | DRB1*04:03 |  | DPB1*04:02 | DQA1*01:05 | DQB1*04:02 |
|  | DRB1*04:04 |  | DPB1*05:01 | DQA1*02:01 | DQB1*05:01 |
|  | DRB1*04:07 |  | DPB1*14:01 | DQA1*03:01 | DQB1*05:02 |
|  | DRB1*07:01 |  | DPB1*15:01 | DQA1*03:02 | DQB1*05:03 |
|  | DRB1*08:01 |  | DPB1*20:01 | DQA1*03:03 | DQB1*06:02 |
|  | DRB1*09:01 |  | DPB1*23:01 | DQA1*04:01 | DQB1*06:03 |
|  | DRB1*10:01 |  |  | DQA1*05:01 | DQB1*06:04 |
|  | DRB1*11:01 |  |  |  |  |
|  | DRB1*12:01 |  |  |  |  |
|  | DRB1*13:01 |  |  |  |  |
|  | DRB1*13:02 |  |  |  |  |
|  | DRB1*14:54 |  |  |  |  |
|  | DRB1*15:01 |  |  |  |  |
|  | DRB1*16:01 |  |  |  |  |

* HLA-DRA alleles derived from SeqPilot software
